# Wavelet Transform Artificial Intelligence Algorithm-Based Data Mining Technology for Norovirus Monitoring and Early Warning

**DOI:** 10.1155/2021/6128260

**Published:** 2021-09-17

**Authors:** Xucheng Fan, Na Xue, Zhiguo Han, Chao Wang, Heer Ma, Yaoqin Lu

**Affiliations:** ^1^Department of Infectious Disease Control, Urumqi Center for Disease Control and Prevention, Urumqi 830026, Xinjiang, China; ^2^Department of Microbiology, Urumqi Center for Disease Control and Prevention, Urumqi 830026, Xinjiang, China

## Abstract

Norovirus monitoring and early warning can be used for diagnosis without etiological testing, and the treatment of this disease does not require the antibiotics. It often occurs in preschool children and affects their growth and development, so the coping measures for this disease are more prevention than treatment. In this study, the clinical data of 2133 children with diarrhea were collected. Based on the artificial intelligence (AI) algorithm of wavelet transform, a related model for data mining and processing of children's intestinal ultrasound images and stool specimens was constructed. Then, the norovirus infection trend was warned based on the wavelet analysis algorithm model. The results showed that the intestinal ultrasound image processed by the wavelet transform algorithm was clearer. The positive detection rate of norovirus in children with clinical diarrhea was as high as 59%, and the children had different degrees of body damage, of which the probability of compensatory metabolic acidosis was the highest. The epidemiological analysis found that children with norovirus infection were mainly concentrated in the age group under 2 years old and over 5 years old and showed a peak of infection in December. In summary, the intelligent algorithm based on wavelet transform can realize the noise reduction of intestinal ultrasound, and it should protect children with susceptible age and susceptible seasons to reduce the clinical infection rate of norovirus.

## 1. Introduction

Norovirus, also known as Norwalk virus, is highly similar to common viruses except for antigenicity [1]. The diarrhea caused by the virus is extremely prevalent, and infection can occur throughout the year. The main population of the disease includes adults and school-age children, with a high incidence in cold seasons [2]. In Western developed countries, more than 80% of all nonbacterial diarrhea outbreaks are infected by the virus every year. Besides, there are similar results in many developing countries [3]. Among children patients with diarrhea under 6 years old in China, the detection rate of norovirus is about 17%, and it has been found that the infection of this virus is also extremely common in the population of China through blood routine investigation [4]. The virus has distinct characteristics such as rapid mutation, strong resistance, and diverse transmission routes. Patients may have clinical manifestations such as diarrhea, vomiting, and fever after illness [5]. According to relevant investigations and studies, norovirus outbreaks have been dominated by other infectious diarrheal diseases in China since 2010. Especially, since the winter of 2014, norovirus outbreaks have increased significantly, higher markedly than previous years, so monitoring and early warning of this virus has become particularly important [6].

Clinical examinations for diarrhea caused by the virus usually use X-ray, endoscopy, B-mode ultrasound, and other examinations, among which B-mode ultrasound has become one of the main examination methods due to its noninvasive and nonradioactive advantages [7]. A large amount of information of the children patients have been recorded by B-mode ultrasound, such as abdominal changes after infection. It is necessary to carry out data mining and summarize the main data information so as to analyze the massive and complex data [8]. However, information deviation is caused due to the influence of noise during B-mode ultrasound examination, so the artificial intelligence algorithm is needed to deal with it [9]. Wavelet transform is a new type of the transform analysis method, which is evolved from the idea of localization of short-time Fourier transform. It overcomes the disadvantage that the window size does not change with frequency and provides a “time-frequency” window, which is an ideal tool to realize signal time-frequency analysis and processing at present [10]. Using the wavelet transform, noise and useful signal can be decomposed to different scales, and wavelet coefficients can be converted, so that useful signal can be distinguished from noise [11]. At present, the application in the science and technology information industry has made remarkable achievements, and it has become a critical part of contemporary scientific and technological work [12]. What is more, its application research in epidemic diseases is relatively small. In order to evaluate the feasibility and effectiveness of data mining technology based on the wavelet transform artificial intelligence algorithm for norovirus monitoring and early warning, wavelet transform artificial intelligence algorithm-based data mining technology was adopted in this study, so as to discuss the diarrhea caused by norovirus infection. Therefore, its feasibility in the monitoring and early warning of virus infection could be obtained, thereby providing reference significance for the application of other diseases.

## 2. Materials and Methods

### 2.1. General Data

2,133 children patients with diarrhea admitted to hospital from April 2016 to June 2020 were selected as the research objects in this study. Among them, 1,154 were boys and 979 were girls, aged 30 days–6 years old after birth, with an average age of 2.13 ± 0.22 years old. The inclusion criteria were as follows: children patients defecated more than 3 times a day and had abnormal changes in fecal characteristics; children patients were younger than 6 years old and did not suffer from other diseases such as pus and blood in the stool and pneumonia; children patients had congenital digestive system diseases; and the family members of the children patients were aware of this study and signed the informed consent forms. The exclusion criteria were as follows: children patients were combined with other basic diseases that affected digestive function; the parents of the children patients did not cooperate with the researcher; the children patients had poor compliance; and the children patients were infected with other epidemic diseases during the research period. Most importantly, the medical ethics committee of hospital reviewed and approved this study.

### 2.2. Methods

#### 2.2.1. Specimen Collection

5 mL of the stool specimen of each included child patient was taken and put into a sterile and dried container. Then, it was placed in phosphate buffer, which was mixed into a 15% stool suspension. Afterwards, the suspension was divided into two evenly. One part was used to detect rotavirus, and the remaining one part was stored in a water tank at a temperature of −80°C to extract deoxyribonucleic acid (DNA) and ribonucleic acid (RNA) from the child patient.

#### 2.2.2. Virus Detection

The norovirus detection kit (produced by Shanghai Fusheng Industrial Co., Ltd.) was adopted in this study, and the detection was carried out based on the operating instructions of the immunochromatography kit. The supernatant was extracted from the stool suspension, which was mixed with the enzyme marker evenly. Next, the mixture was added to the specimen well, which was placed at room temperature and then added with enzyme marker for incubation at room temperature. Finally, the incomplete binding peroxidase was rinsed with lotion, and the results in the kit were observed.

The rotavirus antigen kit (produced by Shanghai Kemin Biotechnology Co., Ltd.) was applied in this study, and the operating instructions of the enzyme-linked immunosorbent assay kit were refereed for detection. The specimens and standard specimens were added in the coated micropores with prereported captured antibodies and washed thoroughly after incubation. Then, the substrate tetramethylbenzidine (TMB) was used for color development. The absorbance value was calculated at 420 nm by using the enzyme-plate method.

### 2.3. Ultrasonic Testing

The Siemens 18L6HD and other color Doppler ultrasound diagnostic equipment was adopted in this study, with the high-frequency probe (7.0–12.0 MHz). Before the examination, the children patients should fast for 3-4 hours, the infants who used milk should fast for more than 4 hours, and the infants and children patients after supplementary food should be fasted for more than 6 hours. During the examination, each child patient was placed in a supine position. The examiner would probe coated with a small amount of the coupling agent and then put on the latex sleeve; in the case of children relaxed, the probe would slowly contact the skin of children, began ultrasound scanning, and carefully observe the stomach and intestinal wall layer changes of children patients.

### 2.4. Analysis of Wavelet Transform Algorithm

The wavelet analysis algorithm belonging to a kind of window size is fixed but the shape can be changed, which is called the “mathematical microscope.” Moreover, it can play its high time resolution and low frequency resolution in the high-frequency part, which shows that the wavelet transform has the self-adaptability to the signal. The wavelet change algorithm was adopted in the denoising of the intestinal ultrasound images of children in this study, so as to provide data for the follow-up exploration of the body damage of children.

In the wavelet transform algorithm, the following can be obtained if *P*(*t*) represents a square cocoa integrable function.

(1) $$\begin{array}{c} P\left(t\right)\in R^{2}\left(e\right). \end{array}$$

In equation (1), *e* stands for a constant, *R* means the upper limit of space, and its Fourier transform *P*(*w*) satisfies the following equation.

(2) $$\begin{array}{c} C_{P}=\int_{-\infty }^{+\infty }\frac{{\left|P\left(w\right)\right|}^{2}}{w}lw<\infty . \end{array}$$

Then, the frequency domain of wavelet transform can be expressed as follows.

(3) $$\begin{array}{c} wt_{i}\left(a,s\right)=\frac{\sqrt{a}}{2\Pi }\int_{-\infty }^{+\infty }i{\left(w\right)}^{P}{\left(awe\right)}^{ws}w. \end{array}$$

In equation (3), *a* represents different transformation scales, the signal to be analyzed is represented by *i*, and *s* indicates the displacement of the fundamental wavelet function.

Continuous wavelet transform is to expand and shift the mother wavelet *P*(*t*) to obtain the function *P*_*a*,*s*_(*t*), which is expressed in the following equation.

(4) $$\begin{array}{c} P_{a,s}\left(t\right)=\frac{1}{\sqrt{a}}P\left(\frac{t-s}{a}\right). \end{array}$$

**Figure 1:**
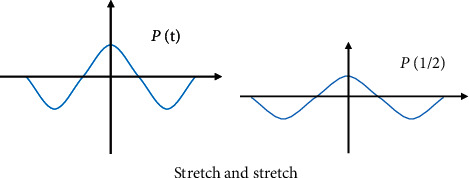
The stretching transformation of wavelet in time domain.

**Figure 2:**
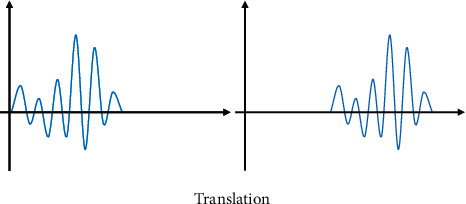
Time shift of wavelet.

In (4), *P*_*a*,*s*_(*t*) is the wavelet basis function that depends on the parameters. The expansion and contraction in the time domain and the time translation can be expressed in Figures 1 and 2.

Discrete wavelet transform usually adopts binary discrete form, as shown in the following equation.

(5) $$\begin{array}{c} a=2^{x}. \end{array}$$

Thus, (6) can be attained.

(6) $$\begin{array}{c} P_{x,s}\left(t\right)=2^{x^{2}}P\left(2^{x}t-s\right). \end{array}$$

The basic principle of wavelet transform noise reduction is presented in the following equation.

(7) $$\begin{array}{c} Y\left(o\right)=G\left(o\right)+e\left(o\right),\;o=1,2,\ldots ,n-1. \end{array}$$

Among them, *Y*(*o*) represents the signal with noise, *G*(*o*) stands for the actual signal, and *e*(*o*) expresses the magnitude of the noise.

**Figure 3:**
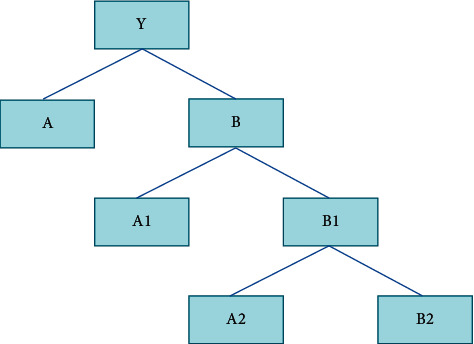
Three-layer decomposition of wavelet transform. A and B stand for weight.

Wavelet transform can decompose signal and noise and separate useful and useless signals. The specific performance is that useful signals have larger decomposition coefficients in some components, while the decomposition coefficients of noise are smaller. In addition, some components have the opposite performance. The decomposition coefficient of noise is large, while the decomposition coefficient of useful signal is small. Finally, the final result can be obtained by coefficient reconstruction after transformation (Figure 3).

The signal-to-noise ratio (SNR), root mean square error (MSE), and carrier-to-noise ratio (CNR) were adopted to evaluate the denoising effect of the algorithm. The calculation equations of SNR, MSE, and CNR are as follows:

(8) $$\begin{array}{c} \text{SNR}=\frac{S}{N0B},\\ \text{MSE}\left(y,\hat{y}\right)=\frac{1}{n}\sum_{i=1}^{n}{\left(y_{i}-\hat{y}\right)}^{2}.\\ \text{CNR}=\frac{S}{N0}. \end{array}$$

In the above equations, *S* represents the signal power, *N*0*B* represents the noise power, and *N*0 represents the noise power spectral density. The higher the SNR, the more prominent the signal and the better the quality of the original signal or transmission information signal. The smaller the MSE value, the better the accuracy of the processed image.

## 3. Results

### 3.1. Processing Performance and Results of Wavelet Transform Algorithm

**Figure 4:**
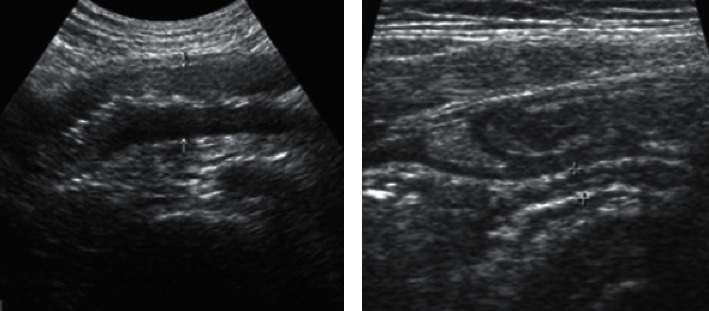
Intestinal ultrasound images of a child patient. (a) The image of a child, female, 10 years old, suffered from abdominal pain and diarrhea for 2 weeks. (b) The image of a child, male, 9 years old, suffered from a tight abdomen, obvious diarrhea, and abdominal pain.

**Figure 5:**
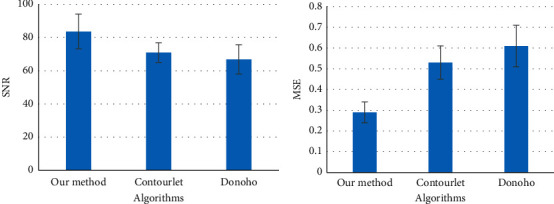
Evaluation of the noise reduction effect. (a) The comparison on SNR. (b) The comparison on MSE.

**Figure 6:**
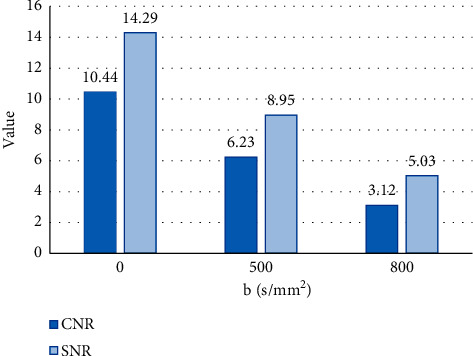
Comparison on SNR of images under different *b* values.

The ultrasound examination results of a child patient with confirmed norovirus infection were selected randomly from the included research objects.  Figure 4 shows that there was inhomogeneous echo in the center of the intestine of this child patient, hypoecho in the outer circle, and thickening of the mesentery at the margin of part of the intestinal wall with enlarged lymph nodes.  Figure 5 shows the results of the noise reduction effect evaluation. Compared with the Contourlet threshold and Donoho threshold, the wavelet transform algorithm used in this study showed a higher SNR value after ultrasonic image denoising and a smaller MSE value. As illustrated in Figure 6, with the increase of the ultrasonic imaging parameter *b* value, the CNR value after wavelet transform denoising was always lower than the SNR value.

### 3.2. Detection Rate of Positive Virus

**Figure 7:**
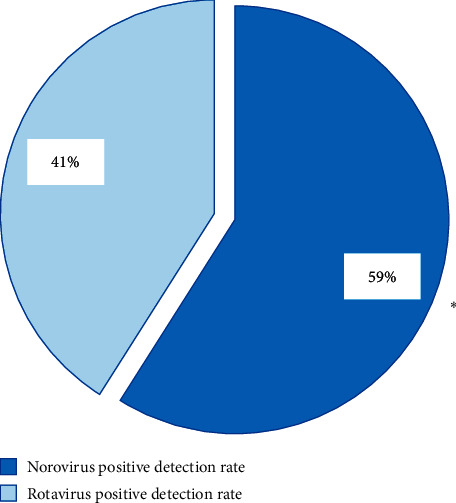
Detection rate of positive virus. ^*∗*^The difference among the groups was statistical obvious (*P* < 0.05).

Figure 7 shows that the positive detection rate of norovirus was higher greatly than the positive detection rate of rotavirus, and the difference was statistically substantial (*P* < 0.05).

### 3.3. Laboratory Test Results of Children Patients with Positive Infections

**Figure 8:**
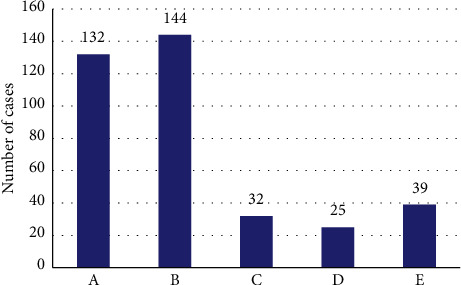
Laboratory test results of children patients with positive infections. (a) Decompensated metabolic acidosis. (b) Compensatory metabolic acidosis. (c) Hypokalemia. (d) Hyponatremia. (e) Liver damage.

Figure 8 shows that children patients with positive infections had different degrees of body damage. Among them, there were 132 cases of decompensated metabolic acidosis, 144 cases of compensatory metabolic acidosis, 32 cases of hypokalemia, 25 cases of hyponatremia, and 39 cases of liver damage.

### 3.4. Age Distribution Results of Children Patients Infected with the Virus and Seasonal Distribution of Virus Infection in Children Patients

Table 1 suggests that the age of children patients infected with the virus was concentrated in children under 2-3 years old and above 5 years old. In addition, the children patients were susceptible to the virus in winter, with the majority in December (Table 2).

## 4. Discussion

Norovirus belongs to a subfamily of Norovirus, namely, the prototype representative strain of human Caliciviridae [13]. Norovirus infections were first detected in China in the early 1990s. Besides, there were outbreaks all over the country since then. In recent years, the infection caused by this virus still shows an increasing trend [14]. The incubation period of the disease is within 1-2 days, which is relatively short [15]. There are two studies comparing the symptoms of diarrhea and vomiting in cases of different age groups [16]. The course of norovirus infection is relatively short, and the duration of clinical symptoms such as diarrhea and fever is only about 3 days. In addition, the patients show poor immunity, and the recovery time after infection is longer [17, 18]. Although the consequences caused by norovirus infection are not very serious, it still cannot be ignored. There are still cases of death caused by infection in clinical practice, and there is also the probability that healthy people will develop into severe cases after being infected with norovirus. Therefore, monitoring and early warning of norovirus has become an essential link.

Wavelet denoising is to eliminate noise through short waves, which is consistent with the basic principle of Gaussian denoising [19]. In recent years, the wave theory has developed rapidly, and it has been widely used in practice due to its good time-frequency characteristics. In new fields, the wave theory has also attracted the attention of many scholars because of its good application effects [20]. In mathematics, denoising is essentially a function approximation problem, that is, how to find the best approximation to the original signal in the function room, and the wave-generating function is expanded and shifted according to the proposed criteria, in order to complete the distinction between the original signal and the noise signal. In other words, the best mapping is found from the actual signal space to the wave function space. From the point of view of signal science, denoising is a problem of signal filtering. Although denoising can be regarded as low-pass filtering to a large extent, it is better than the traditional low-pass filtering in retaining signal features completely after processing [21]. Thus, it is found that wavelet denoising is actually a combination of feature extraction and low-pass filtering. Through collecting a large number of cases, all the research objects underwent the ultrasound examinations in this study, and the results were processed by the wavelet transform algorithm, so the following conclusions were drawn. The positive detection rate of norovirus was higher than that of rotavirus. The age of children patients infected with norovirus was concentrated in children under 2-3 years old and above 5 years old, and the children were susceptible to norovirus in winter and most of them were in December. From this, the susceptible population and susceptible season of norovirus could be known.

Data mining technology is to obtain valuable knowledge and rules that users may be interested in from a large amount of data in the storage space, so as to tap out the potential of users [22]. These rules can provide useful information for business decisions and financial forecasts. In 1990, with the wide application of the network system and the rapid development of network technology, database technology has stepped into a new stage of management of graphs and images from the management of some simple data in the past, and the amount of data is also increasing [23]. According to the analysis, data mining is mainly carried out from the four aspects of classification, estimation, prediction, and clustering. Therefore, it is necessary to make sufficient preparations for the purpose of the operation and data collection before the operation. In this study, the ultrasound examination results and virus detection results of a large number of children patients were collected and analyzed, so as to construct the correlation model of related feature transformation. What is more, the data acquisition was mainly to prepare for the subsequent data mining. When data mining is performed, it is necessary to select data from the dataset and select the data needed to build the model from the original dataset. In this study, the data mining results found that the early ultrasound examinations of children patients in the susceptible age group showed slight intestinal edema or effusion and slight changes in gastrointestinal function, which affected the diet and rest of children. Some children patients might have clinical manifestations of vomiting, which was basically in line with the observation results of clinicians.

## 5. Conclusion

In this study, the main focus was the application of monitoring and early warning detection technology of norovirus, namely, data mining technology. Therefore, a large number of children patients with diarrhea symptoms were selected, and the ultrasonic examination results were collected. Meanwhile, the wavelet transform algorithm was applied to denoise processing of ultrasonic images to facilitate the normalization and collation of data. The research results showed that the ultrasound images processed by the wavelet transform algorithm had higher quality, which was convenient for doctors to diagnose the diseases of children in combination with the image data. Based on the results of data mining technology analysis of children infected with norovirus, it was found that in clinical practice, children with diarrhea were more likely to be infected with norovirus than rotavirus, and children younger than 2 years old or older than 5 years old are more susceptible to norovirus in December. In summary, data mining technology based on the wavelet transform artificial intelligence algorithm had a relatively accurate warning effect and could also predict the future trend of disease data, so it could be promoted and applied clinically. The disadvantage of this study is that the presentation of case data is less, which makes the research results lack of intuitive data support. Therefore, further research and analysis are needed to provide more effective clinical reference value.

## Figures and Tables

**Table 1:** Age distribution results of children patients infected with the virus (cases).

Age distribution (years old)	Positive norovirus	Positive rotavirus
<1	211	214
1-2	204	131
2-3	122	83
3-4	79	60
4-5	65	54
>5	162	47

**Table 2:** Month distribution of virus infection in children patients (cases).

Month	Positive norovirus	Positive rotavirus
1	11	87
2	10	97
3	12	99
4	10	13
5	6	14
6	8	7
7	10	12
8	17	13
9	75	17
10	132	21
11	241	96
12	311	113

## Data Availability

The data used to support the findings of this study are available from the corresponding author upon request.
